# The Surgical Challenges of Intertrochanteric Hip Fracture in a Patient with Polio Dysplastic Hip and Previous Distal Femur Fracture: A Case Report

**DOI:** 10.5704/MOJ.2203.021

**Published:** 2022-03

**Authors:** MC Lai, SHA Ng, AXR Premchand

**Affiliations:** Department of Orthopaedic Surgery, Khoo Teck Puat Hospital, Singapore

**Keywords:** poliomyelitis, hip fracture, dysplastic hip, cephalomedullary nail

## Abstract

Poliomyelitis is on the verge of eradication since the introduction of the vaccine in 1950. In developed countries, those afflicted with the disease are primarily in their sixth decade and beyond, usually with disabling complications. Due to the diminished muscle power coupled with the abnormal bony anatomy and joint contractures, patients with polio present unique surgical challenges when they sustain fragility fractures. We report an uncommon case of intertrochanteric hip fracture in a limb affected with polio and hip dysplasia, on a background of ipsilateral distal femur fracture with previous surgical fixation. We aim to outline the challenges encountered during the surgery and the preoperative planning to overcome these shortcomings.

## Introduction

Due to global vaccination efforts, the number of newly diagnosed polio cases have fallen dramatically, and the disease is on the verge of eradication from most of the world1. However, an estimated population of 10 – 20 million polio survivors are still living with the disabling consequences of this disease^1^. This groups of patients proceed to develop paralysis of lower extremities, characterised by flaccid muscle tone, asymmetric involvement, and limb length discrepancy. Most of the polio patients in the developed countries are well into their sixth and seventh decades and prone to osteoporotic fractures^2^.

We report a case of intertrochanteric hip fracture with concomitant dysplastic acetabulum in a post-polio patient and the challenges faced during surgical management.

## Case Report

A 71-year-old Chinese lady was admitted to our institution after a fall and was subsequently unable to weightbear due to a painful left hip. This patient has a background history of childhood poliomyelitis but was ambulant in the community with the aid of a walking stick and independent with her activities of daily living (ADLs). She also has a history of osteoporosis for which she was prescribed oral bisphosphonates. In addition, this patient also previously sustained a left distal femur fracture two years ago and underwent an open reduction and internal fixation with plate osteosynthesis. This, however, was complicated by non-union and required revision surgery with bone grafting before union.

**Fig. 1:**
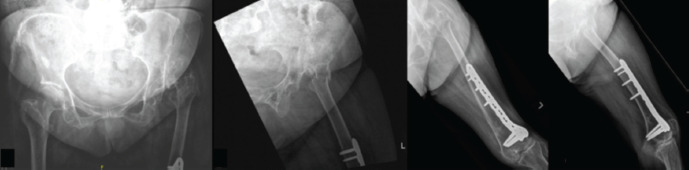
Pre-operative radiographs showed a left hip intertrochanteric fracture and concurrent left dysplastic hip. The left femur radiographs showed left distal femoral plate proximally was more anterolateral due to altered femoral anatomy.

On examination, the patient had pain over her left hip. Her left lower limb demonstrated muscle atrophy mainly over the quadriceps muscles. Her left foot also showed an equinus contracture deformity. There was reduced tone and deep tendon reflexes were hyporeflexic in the affected limb. The power for myotomes L2 to S1 were reduced with a Medical Research Council (MRC) grade of 3 to 4. The myotome L4 was not assessed due to the contracture. Her sensation was intact. Her pelvis and hip radiographs demonstrated a left hip 3 part intertrochanteric fracture (Fig. 1). A dysplastic hip with superior subluxation of the left femoral head was also noted. Coxa valga was demonstrated with a measured neck shaft angle of 150°. Left femur radiographs performed showed the previous distal femur fixation. The left femur was hypoplastic, in keeping with the history of poliomyelitis. A computed tomography (CT) scan of the left hip was performed to evaluate the bone stock and anatomy, which demonstrated the anteversion of the proximal femora.

She was counselled for surgical fixation due to her good premorbid status. During the pre-operative templating, we chose a cephalomedullary device for fixation, but the presence of increased anteversion and valgus of the proximal femora and a hypoplastic femur with its narrow medullary canal made surgical fixation challenging. In addition, the previous distal femur plate osteosynthesis made intramedullary nail (IMN) fixation difficult. With these factors in mind, the definitive surgical plan was to perform an IMN fixation while circumventing the challenges mentioned.

**Fig. 2:**
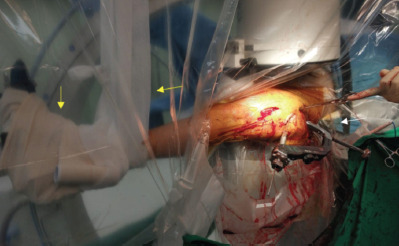
Elastoplast used to secure the foot and a sling was applied to prevent the foot from slipping off the foot traction (yellow arrows). The incision for entry point was more proximal and posterior to get centre-centre blade position (white arrow).

Under general anaesthesia, the patient was positioned supine on a traction table. The patient’s severe equinus contracture of her left foot made intra-operative positioning challenging as it was difficult to secure her foot in the stirrup. Tensoplast® tape around the distal leg and a sling made of cotton bandage was applied around the calf to hold her left lower limb in place (Fig. 2).

Initial closed reduction was performed under the guidance of image intensifier. The incision for the entry point was made more posteriorly to facilitate a centre-centre position of the helical screw in the femoral head-neck in view of the anteversion of the femoral neck. A second 5mm stab incision was made to introduce a roberts artery forceps to further improve the reduction. Three Kirschner wires were then used to hold the reduction. Two proximal screws from the previous distal femur plate were removed prior to intramedullary nail insertion. A size 9, 200mm Proximal Femoral Nail Antirotation (DePuy Synthes, PFNA) was measured for this patient. Two unicortical screws were reinserted into the distal femur plate to replace those removed earlier.

**Fig. 3:**
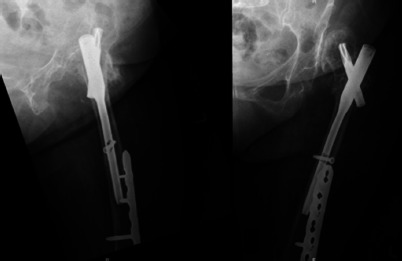
Post-operative radiographs showed the left hip fracture has well healed. No evidence of implant loosening.

The patient was allowed to weightbear after surgery. Her recovery was uneventful, and she was transferred to a community hospital for rehabilitation a week later. At six months follow-up, she could ambulate with a walking frame. Her latest radiographs showed that the fracture has healed well (Fig. 3).

## Discussion

Fracture fixations in polio patients are challenging for the orthopaedic surgeon. The bone of the affected limb is often hypoplastic, osteopaenic, deformed, paralysed and wasted^3^. The hips may also be associated with excessive anteversion, a valgus neck and hypoplasia which pose difficulties in the subsequent surgical management^4^.

In our case, three main challenges were encountered during surgery. The first was the left foot and ankle severe equinus contracture which caused difficulty during positioning on the traction table. These patients may also present with knee flexion and hip flexion abduction deformities^3^. The reduction methods depends on the degree of joint deformities and type of contractures. Manual traction in general should be sufficient to reduce the fracture. However, strong traction should be avoided as it may lead to displacement or over distraction of the fracture fragments due to limb flaccidity in polio patients. Open reduction was another possible option, but this method requires an incision with risks of soft tissue disruption, intra-operative blood loss and possible neurovascular injuries. In this case, we used elastic tapes and a sling to secure the foot to prevent it from slipping off the traction table.

The second challenge of this case was the abnormal bony anatomy. The affected hip was excessively anteverted with the presence of coxa valga. In this case, more internal rotation was necessary to achieve a satisfactory reduction.

Since the proximal femur is anteverted and subluxed superiorly due to the dysplastic hip, a more proximal and posterior entry point was made^4^. We also had to countersink the nail more than usual in order to achieve a centre position of the helical blade in the femoral head due to its increased neck shaft angle.

The type of implant for intertrochanteric fracture is based on the fracture pattern and its inherent stability. Most of these fractures are treated with intramedullary nails or sliding hip screws. Patients with polio may have a narrow intramedullary canal, which poses difficulty for IMN insertion^5^. Moreover, the presence of the valgus neck complicates the surgical fixation. Nonetheless, we managed to insert a cephalomedullary device in this patient as our preoperative templating.

The last challenge of this case was the distal femur plate from her previous surgery. The plate can potentially interfere with the distal interlocking screw of the nail and therefore necessitates removal. If the nail is too short, it will potentially cause a stress riser at the interface between the tip of the nail and the proximal plate. There were a few alternatives which were formulated and discussed. Our first option was to remove the distal femur implants completely followed by insertion of the cephalomedullary device, but this was an extensive procedure and deemed unnecessary. Our second option was to proceed with the cephalomedullary device without inserting a distal interlocking screw, but we did not wish to compromise on distal rotational stability with a potential risk of developing stress risers, so this was not chosen. Also, the position of the distal femur plate was anterolateral, so it did not obstruct the insertion of the distal locking nail. The last alternative was to use a femur nail with the anteroposterior distal interlocking screw, but this ran the risk of injuries to the anterior neurovascular bundle in view of the altered anatomy.

The reported post-operative complications of intertrochanteric hip fractures include non-unions, malunion, peri-implant fractures, cut-out and irritation due to prominent implant, which can leads to a re-operation rate up to 16%^2^. For our case, she had none of these complications. At six months follow-up, she had returned to her premorbid status with radiographs showing complete fracture healing (Fig. 3).

In summary, there are numerous challenges in treating fractures in patients with polio. It is imperative that the surgeon identify the challenges faced to formulate a detailed pre-operative plan to counter the problems associated with implant placement. Multidisciplinary management is mandatory and early post-operative rehabilitation is essential to ensure a good surgical outcome.
